# Detection of emerging antibiotic resistance in bacteria isolated from subclinical mastitis in cattle in West Bengal

**DOI:** 10.14202/vetworld.2017.517-520

**Published:** 2017-05-12

**Authors:** Arnab Das, Chanchal Guha, Ujjwal Biswas, Partha Sarathi Jana, Amaresh Chatterjee, Indranil Samanta

**Affiliations:** 1 Animal Resources Development Department, Institute of Animal Health and Veterinary Biologicals, Government of West Bengal, Kolkata, West Bengal, India; 2 Department of Veterinary Epidemiology and Preventive Medicine, Faculty of Veterinary and Animal Sciences, West Bengal University of Animal and Fishery Sciences, Kolkata, West Bengal, India; 3 Department of Veterinary Microbiology, Faculty of Veterinary and Animal Sciences, West Bengal University of Animal and Fishery Sciences, Kolkata, West Bengal, India

**Keywords:** antibiotic resistance, cattle, *Escherichia coli*, India, sub-clinical mastitis

## Abstract

**Aim:**

The aim of this work was to detect antibiotic resistance in Gram-negative bacteria isolated from subclinical mastitis in cattle in West Bengal.

**Materials and Methods:**

The milk samples were collected from the cattle suffering with subclinical mastitis in West Bengal. The milk samples were inoculated into the nutrient broth and incubated at 37°C. On the next day, the growth was transferred into nutrient agar and MacConkey agar. All the pure cultures obtained from nutrient agar slant were subjected to Gram-staining and standard biochemical tests. All the bacterial isolates were tested in vitro for their sensitivity to different antibiotics commonly used in veterinary practices. All Gram-negative isolates including positive control were subjected to polymerase chain reaction (PCR) for detection of bla_CTX-M_, bla_TEM_, bla_SHV_, bla_VIM_, tetA, tetB, tetC, and tetM genes considered for extended-spectrum β-lactamase (ESBL), metallo-β-lactamase, and tetracycline resistance.

**Results:**

In total, 50 Gram-negative organisms (Escherichia coli, Proteus, Pseudomonas, Klebsiella, and Enterobacter) were isolated from milk samples of subclinical mastitis infected cattle. Among these Gram-negative isolates, 48% (24/50) were found either ESBL producing or tetracycline resistant. Out of total 50 Gram-negative isolates, bla_CTX-M_ was detected in 18 (36%) isolates, and 6 (12%) harbored bla_TEM_ genes in PCR. None of the isolates carried bla_SHV_ genes. Further, in this study, 5 (10%) isolates harbored tet(A) gene, and 8 (16%) isolates carried tet(B) gene. No tet(C) gene was detected from the isolates.

**Conclusion:**

This study showed emerging trend of antibiotic-resistant Gram-negative bacteria associated with subclinical mastitis in cattle in West Bengal, India.

## Introduction

Subclinical mastitis is the most fatal infection confronted by the dairy industry today with significant economic losses worldwide including India [1]. It also poses a major public health risk due to transmission possibility of zoonotic bacteria or their toxin along with antibiotic resistance genes [2]. Most of the subclinical mastitic animals do not produce characteristic symptoms of mastitis and are persistent shedder of zoonotic bacteria without adequate awareness of farmers regarding the transmission possibility [3].

Subclinical mastitis is considered to have a multifactorial etiology including several groups of microorganisms such as bacteria, virus, fungi, yeast, and algae [4]. Among the Gram-negative bacterial etiological agents, the major group includes coliform bacteria (*Escherichia coli*, *Enterobacter*, *Klebsiella*), *Pseudomonas* and *Serratia* [5].

Early detection of subclinical mastitis and dry cow therapy with proper antibiotics, use of post-milking teat disinfectants and effective pre-milking hygiene becomes relevant to minimize economic losses in dairy farms and to prevent zoonotic transmission [6]. However, the etiological and commensal bacteria present in animals and exposed to the antimicrobial pressure, develop survival strategies through evolutionary adaptations [7]. Gram-negative bacteria specially *Enterobacteriaceae* organisms mostly produce β-lactamase enzymes to prevent the action of β-lactam antibiotics. There are more than 1000 β-lactamase enzymes that can be classified under four main classes, i.e., A-D [8]. The most clinically important Class A enzymes, found in *Enterobacteriaceae*, are known as extended-spectrum β-lactamases (ESBLs). It can confer resistance to a variety of β-lactam ­antibiotics, including penicillins, 2^nd^, 3^rd^ and 4^th^-generation cephalosporins and monobactams (e.g., ­aztreonam), but usually not the carbapenems or the cephamycins (e.g., cefoxitin). There are three classical ESBLs, i.e., TEM (except TEM-1), SHV (except SHV-1 and 2), and CTX-M [9]. Among them, CTX-M is observed as the most prevalent type worldwide [10]. Other than ESBL genes, possession of tetracycline resistance gene is also common in Gram-negative bacteria [11].

In India, subclinical mastitis in cattle and buffaloes caused by Gram-positive bacteria such as *Staphylococcus* spp. are reported from different states such as Karnataka, Punjab, and others [3,12]. However, etiological correlation of Gram-negative bacteria with subclinical mastitis in Indian cattle is less explored. This study was conducted to know the occurrence of Gram-negative bacteria in subclinical mastitis in cattle associated with antibiotic resistance potential.

## Materials and Methods

### Ethical approval

The study was approved by Instituional Animal Ethics Committee, WBUAFS.

### Sampling

The udders of the suspected animals were examined for fibrosis, inflammatory swellings, visible injury, tick infestation, atrophy of the tissue, and swelling of supramammary lymph nodes. The size and consistency of mammary quarters were inspected for the presence of any abnormalities, such as ­disproportional symmetry, swelling, firmness, and blindness. Information relating to the previous health history of the mammary quarters and causes of blindness was obtained from the owners of the farm. The mastitic milk was collected aseptically as described earlier [13]. The collected milk samples were transported to the laboratory maintaining the cold chain.

### Isolation of Gram-negative bacteria

Initially, the milk samples were inoculated into the nutrient broth (HiMedia, India) and incubated at 37°C. On the next day, the growth was transferred into nutrient agar and MacConkey agar (HiMedia, India). The convex glistening single colonies with greenish discolouration in nutrient agar and the pink and pale colored colonies in MacConkey agar were isolated into nutrient agar slant as a pure culture. All the pure cultures obtained from nutrient agar slant were subjected to Gram-staining and standard biochemical tests as described earlier [14].

### Antibiotic sensitivity test

All the bacterial isolates were tested *in vitro* for their sensitivity to different antibiotics commonly used in veterinary practices. The antibiotic disks oxytetracycline, amikacin, gentamicin, amoxicillin and clavulanic acid, amoxicillin sulbactam, ceftriaxone and sulbactam, ceftriaxone and tazobactam, enrofloxacin, ceftriaxone and cefotaxime (HiMedia) were selected for the study. The interpretation was done in accordance to performance standards for antimicrobial disks susceptibility tests, Clinical Laboratory Standard Institute [15].

### Polymerase chain reaction (PCR)-based detection of ESBL and tetracycline resistance genes

For PCR-based detection of major ESBL genes *(bla_TEM_, bla_SHV_, bla_CTX-M_)* and tetracycline resistance (*tet(A), tet(B)* and *tet(C)*) genes from all the bacterial isolates, DNA was extracted as per the method described by Bonnet *et al*. [16]. All Gram-negative isolates including positive control were subjected to PCR for detection of *bla_CTX-M_*, *bla_TEM_*, *bla_SHV_*, *bla_VIM_*, *tetA, tetB, tetC*, and *tetM* genes considered for ESBL, metallo-β-lactamase, and tetracycline resistance. The PCR was performed in a thermocycler (BioRad, USA) with the primers (Imperial life sciences, India) and the cycle conditions as described earlier [16].

## Results

In this study, 50 Gram-negative organisms (*E. coli, Proteus*, *Pseudomonas*, *Klebsiella*, and *Enterobacter*) were isolated from milk samples of subclinical mastitic cattle. Among these Gram-negative isolates, 48% (24/50) isolates were detected phenotypically as either ESBL producing or tetracycline resistant in antibiotic sensitivity test.

**Figure-1:**
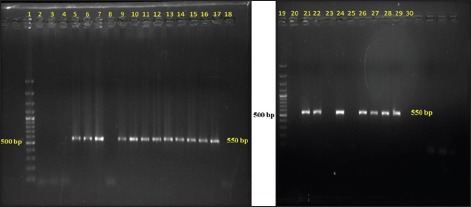
Gel electrophoresis image of polymerase chain reaction amplified products of *bla_CTX-M_* gene of Gram-negative isolates; Lane 1 - 1500 bp DNA ladder; Lane 2-14, 16-19 - Isolated *Escherichia coli* samples; Lane 15 - Positive control for *bla_CTX-M_* gene; Lane 20 - Negative control.

**Figure-2:**
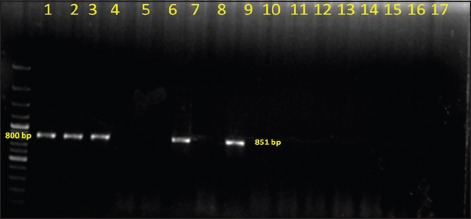
Gel electrophoresis image of polymerase chain reaction amplified products of *bla_TEM_* gene of Gram-negative isolates; Lane 1 - 1500 bp DNA ladder; Lane 2-8, 10-16 - Isolated *Escherichia coli* samples; Lane 15 - Positive control for *bla_TEM_* gene; Lane 20 - Negative control.

**Figure-3:**
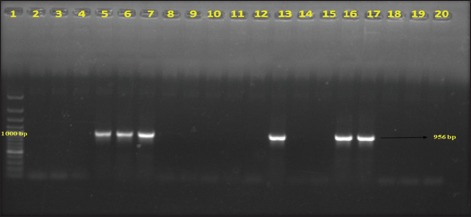
Gel electrophoresis image of polymerase chain reaction amplified products of *tetA* gene of Gram-negative isolates; Lane 1 - 1500 bp DNA ladder; Lane 2-16, 18-19 - Isolated *Escherichia coli* samples; Lane 17 - Positive control for *tetA* gene; Lane 20 - Negative control.

**Figure-4:**
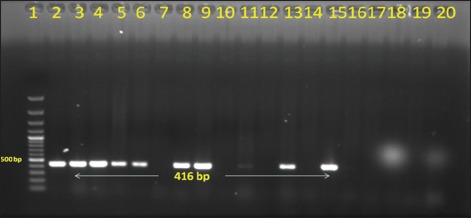
Gel electrophoresis image of polymerase chain reaction amplified products of *tetB* gene of Gram-negative isolates. Lane 1 - 1500 bp DNA ladder; Lane 2-14, 16-19 - Isolated *Escherichia coli* samples; Lane 15 - Positive control for *tetB* gene; Lane 20 - Negative control.

Out of total 50 Gram-negative isolates, *bla*_*CTX-M*_ was detected in 18 (36%) isolates, and 6 (12%) harbored *bla_TEM_* genes in PCR (Figures-1 and 2). None of the isolates carried *bla_SHV_* genes. Further, in this study, 5 (10%) isolates harbored *tet(A)* gene and 8 (16%) isolates carried *tet(B)* gene (Figures-3 and 4). No *tet(C)* gene was detected in the isolates.

## Discussion

Subclinical mastitis is the most fatal infection confronted by dairy industry today with significant economic losses worldwide including India [1]. Moreover, with the extensive use of β-lactam antibiotics, cattle and other ruminants represent a considerable source for the transmission of antibiotic resistance genes (e.g., ESBL) or antibiotic resistant strains to the human intestinal bacterial flora [17]. This study was conducted to know the occurrence of Gram-negative bacteria in subclinical mastitis in cattle associated with antibiotic resistance potential.

Among these Gram-negative isolates, 48% (24/50) were either ESBL producing or tetracycline resistant in this study. Earlier reports from different countries revealed the lower prevalence of ESBL-producing *Enterobacteriaceae* isolates (0.4-13%) associated with bovine mastitis [18-20]. In India, few reports are available regarding detection of ESBL/New Delhi metallo-β-lactamase-producing *E. coli* in milk samples collected from clinical or subclinical mastitic cattle [17,21]. The prevalence study of ESBL-producing Gram-negative bacteria with substantial numbers of mastitic milk samples is not apparently available in India to compare the present finding. However, the study indicates about alarming rise in the occurrence of ESBL-producing Gram-negative isolates in subclinical mastitic cattle.

Out of total 50 Gram-negative isolates, *bla*_*CTX-M*_ was detected in 18 (36%) isolates, and 6 (12%) harbored *bla_TEM_* genes in PCR. None of the isolates carried *bla_SHV_* genes. At present, CTX-M is the major ESBL enzyme produced by different clonal complexes of *Enterobacteriaceae* which mostly replaced the SHV and TEM-type ESBLs during the last decade [22]. It was also observed that CTX-M and TEM were the most prevalent bla-encoded enzyme in human clinical isolates worldwide [23-25]. As well as, CTX-M ESBL producing *Klebsiella pneumonae* was also isolated from the cases of bovine mastitis [26].

Further, in this study, 5 (10%) isolates harbored *tet(A)* gene and 8 (16%) isolates carried *tet(B)* gene. No *tet(C)* gene was detected from the isolates. Earlier studies indicated the presence of *tet* genes in Gram-negative bacteria isolated from bovine mastitis and other infections [27,28].

## Conclusion

The present study showed emerging trend of antibiotic resistant gram negative bacteria associated with sub-clinical mastitis in cattle in West Bengal, India.

## Authors’ Contributions

AD: Conducted study. CG, UB, PSJ, AC and IS: Planned the study. AD and IS: Drafted and revised the manuscript. All authors read and approved the final manuscript.
